# Analysis on Three-Dimensional Strength Influencing Factors and Control Measures of Asphalt Mixtures

**DOI:** 10.3390/ma13112541

**Published:** 2020-06-03

**Authors:** Tuo Huang, Mi Li, Mou-ying Huang, Hao-hao Jiang, Yao Tang, Hong-fu Liu

**Affiliations:** 1 National Engineering Laboratory for Highway Maintenance Technology, School of Traffic and Transportation Engineering, Changsha University of Science & Technology, Changsha 410114, China; ht@csust.edu.cn; 2 School of Traffic and Transportation Engineering, Changsha University of Science & Technology, Changsha 410114, China; 18101020077@stu.csust.edu.cn (M.L.); 13046264559@stu.csust.edu.cn (M.-y.H.); jhh1993@stu.csust.edu.cn (H.-h.J.); tangyao@stu.csust.edu.cn (Y.T.)

**Keywords:** asphalt mixture, various factors, unified three-dimensional strength calculation model, orthogonal test, strength regulation

## Abstract

Strength is an important parameter for the design of asphalt pavement materials and structures. To study the influence of various factors on the three-dimensional strength of asphalt mixtures, three aggregate gradations (dense-graded bituminous mixture AC-13, stone mastic asphalt SMA-13 and bituminous stabilization aggregate paving mixture OGFC-13) and two binders (SBS modified bitumen and 70# base bitumen) were used to prepare the asphalt mixture specimens. Among them, SBS+SMA-13 asphalt mixture has the best performance. On this basis, the uniaxial compressive test, uniaxial tensile test and confining triaxial test were conducted on the SBS+SMA-13 asphalt mixture under six oil-stone ratios conditions (5.5%, 5.7%, 5.9%, 6.1%, 6.3%, and 6.5%), six temperatures conditions (5 °C, 10 °C, 15 °C, 20 °C, 25 °C, and 30 °C), and five loading rates conditions (1 mm/min, 2 mm/min, 3 mm/min, 4 mm/min, and 5 mm/min). In addition, a unified three-dimensional strength calculation model considering the influence of temperature, loading rate, and oil-stone ratio was proposed, and the change law of the three-dimensional strength with these above factors was revealed. Furthermore, two sets of three-factor three-level orthogonal tests were carried out on the SMA-13 asphalt mixture. The sensitivity analysis and strength regulation research between three-dimensional strength and each factor were carried out. The results show that the type of asphalt has the greatest influence on the strength of the mixture, the temperature has the second most influence, the loading rate has less influence, and the oil-stone ratio has the least influence. The strength regulations proposed to improve the strength of the asphalt mixture include the use of modified asphalt, high-temperature stability high-quality asphalt, and the lower oil-stone ratio than the Marshall optimal oil-stone ratio. The strength control measures proposed from the perspective of the three-dimensional stress state, the joint failure of each stress components and real stress states are taken into consideration.

## 1. Introduction

Asphalt pavement is currently the most widely used high-grade pavement structure form around the world [1], for its performance, convenience in construction and maintenance, evenness, and comfort when driving. As the main road material, the mechanical properties of asphalt mixtures, especially direct tensile, uniaxial compression, and confining triaxial strength characteristics are quite important parameters for the design of mixture composition and the combined design of asphalt pavement structures [2]. Asphalt mixtures strength parameters are important parameters for pavement design [3]. Therefore, it is of great significance to study the factors affecting the strength of asphalt mixtures [4] and put forward corresponding control measures to ensure the quality and durability of asphalt pavement [5,6,7].

At present, the research on the strength characteristics of asphalt mixtures mostly focuses on the traditional experiments of tension, compression, bending, shear, and torsion [8,9,10]. As a typical visco-elastoplastic material, the difference of its mechanical properties is directly related to the stress state and test conditions [11]. Kim [12], Lee [13], and Pszczola [14] carried out the strength characteristics of the asphalt mixture under uniaxial tension, uniaxial compression, indirect tension, and semicircular bending under different temperatures and loading speeds which combined with molecular dynamics [15] and fracture mechanics [16]. Some researchers, such as Habeeb [17], Liu [18], Ranieri [19], and Ge [20] have carried out research on the effects of gradation, asphalt type, asphalt content and other factors on the strength performance of the mixture, and proposed the regulation measures to the strength of asphalt mixture [21,22,23]. These research are of reference significance.

Asphalt pavement structure is in a typical three-directional tension-compression combined stress state under the combined effect of temperature and vehicle load. It is obviously that triaxial test can better simulate the stress state of pavement structure [24]. For this, Huang et al. [25] developed a triaxial test equipment, carried out the three-directional loading test, and established an octahedral failure criterion model under complex stress state. To facilitate the engineering design and promote the application, a method of establishing a three-dimensional strength model through conventional direct tensile, uniaxial compression, and confining triaxial tests is further proposed to consider the combined failure effect between each stress components.

**Figure 1:**
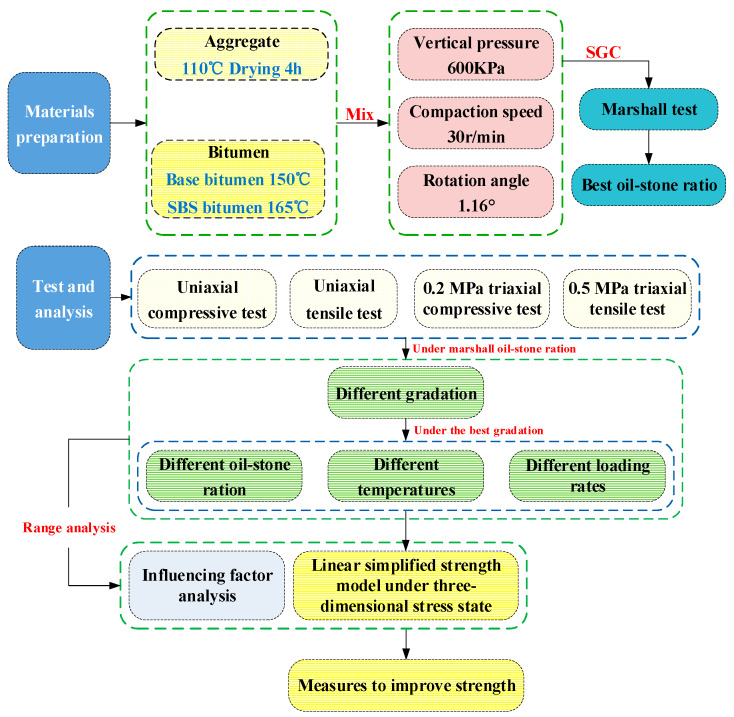
The research program of the failure strength models of asphalt mixtures under various factors.

Therefore, this article uses the above method to test the widely used SMA-13 asphalt mixture and establishes a unified three-dimensional strength calculation model that considers the effects of temperature, loading speed, and oil-stone ratio, and analyzes the influence of various factors through orthogonal experiments. The three-dimensional strength control measures are proposed in order to make better use of the strength potential of the asphalt mixture. The research scheme is shown in Figure 1.

## 2. Materials and Methods

### 2.1. Materials and Specimens Preparation

**Figure 2:**
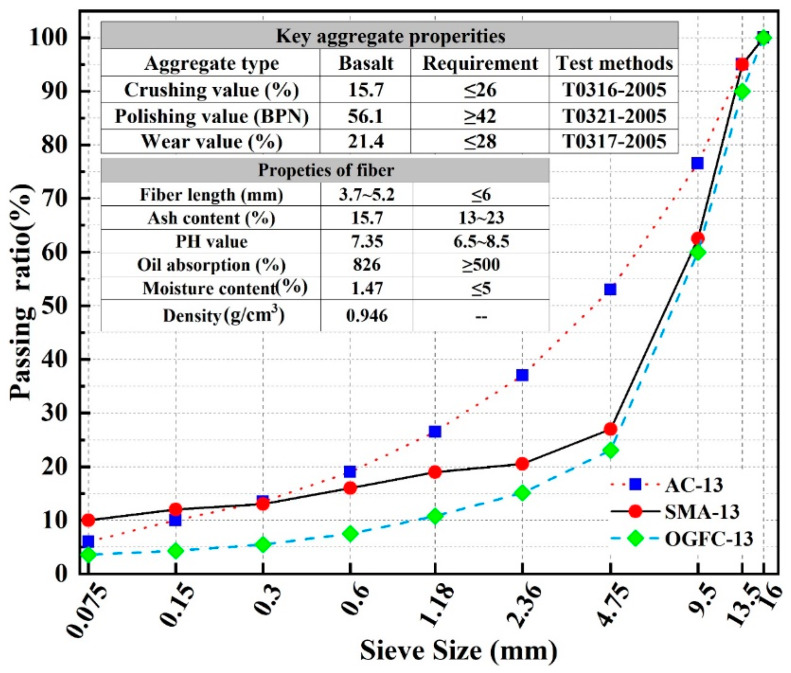
The gradation of three type of asphalt mixture.

It has been clarified that to study the influence of gradation and asphalt type on the three-dimensional strength of asphalt mixture, the Continuous-dense-graded bituminous mixture AC-13, Gap-dense-graded bituminous mixture SMA-13 and Gap-open-graded bituminous paving mixture OGFC-13 were used in this research, which were widely used in asphalt surface layer. The gradations are shown in Figure 2. In addition, the aggregate type is basalt, the apparent density and mechanical properties are shown in Figure 2 and Table 1. The binder is widely used SBS (Styrene-Butadiene-Styrene Block Copolymer) modified bitumen and 70 # base bitumen, and its properties are shown in Table 2. The wood fiber was used in SMA-13 and OGFC-13asphalt mixture. The fiber content is 0.5%, the basic properties are shown in Figure 2. 

**Figure 3:**
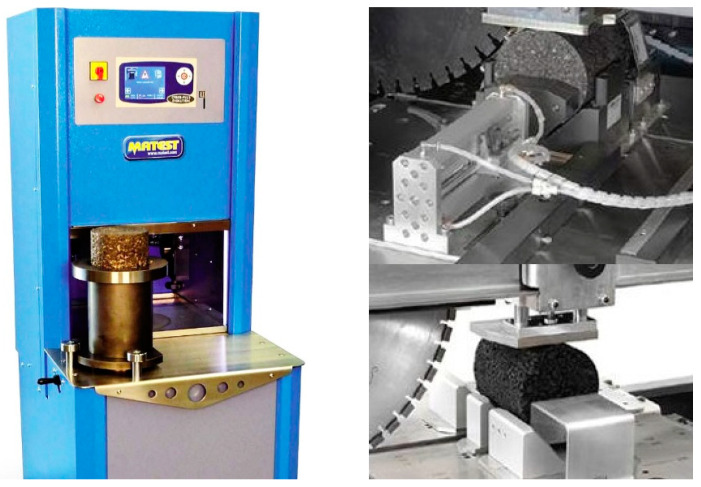
Gyratory compactor device and test specimens.

The optimal oil-stone ratio for the three gradations was determined according to the Marshall test. The Marshall test results are shown in Table 3. Rotary compaction was used to form a cylindrical test piece with a diameter of 100 mm and a height of 106 mm [26]. The surface of the specimen was polished with a diamond blade, and a cylindrical specimen with a diameter of 100 mm and a height of 100 mm was obtained, as shown in Figure 3.

### 2.2. Testing Conditions and Procedures

#### 2.2.1. Testing Plan

**Figure 4:**
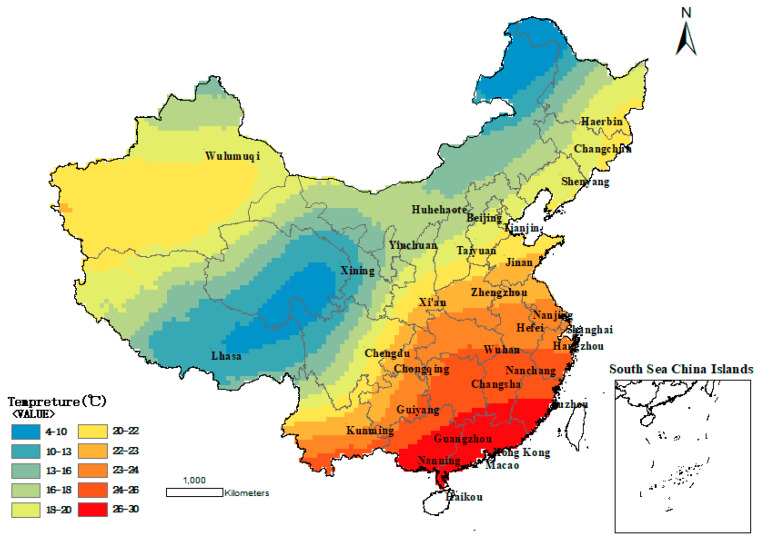
China benchmark equivalent temperature map.

In the *Specifications for the design of highway Asphalt pavement* (JTG D50-2017), the influence of temperature was emphatically considered in the damage analysis of the reference pavement structure. To consider the influence of temperature, the pavement structure damage under different air temperature conditions is converted to the equivalent damage under standard temperature (20 °C). The equivalent temperature is based on the hottest monthly average temperature, the coldest monthly average temperature, and the annual average temperature for 10 consecutive years in the specification. According to the benchmark equivalent temperature $T_{\xi }$ in the specification, the equivalent temperature distribution map of each region in China can be obtained by ArcGIS software using interpolation processing, as shown in Figure 4. Therefore, 5 °C, 10 °C, 15 °C, 20 °C, 25 °C, and 30 °C were selected as the experimental temperatures to better simulate the actual working temperature of the road. The specimens should be placed in the temperature control box for more than 4h before the test to ensure the accuracy of the test temperature. 

In the *Standard Test Methods of Bitumen and Bituminous Mixtures for Highway Engineering* (JTG E20-2011), the loading rate of cylinder uniaxial compression test is 2 mm/min. To analyze the impact of loading rate, the values near the standard loading speed were selected for testing, i.e., the uniaxial compression, direct stretching and confining triaxial tests were carried out with the loading rate of 1 mm/min, 2 mm/min, 3 mm/min, 4 mm/min, and 5 mm/min. 

The strength test of three gradations (AC-13, SMA-13, OGFC-13) of asphalt mixture were performed under the Marshall optimal oil-stone ratio respectively. The results showed that SMA-13 asphalt mixture had the best performance, thus the strength test under different oil-stone ratio also conducted on SMA-13 asphalt mixture. According to the Technical Specifications for Construction of Highway Asphalt Pavement (JTG F40-2004), the asphalt content should be increased 0.1~0.3% at the cold area or decreased 0.1~0.5% at the hot area for adopt the traffic conditions. In addition, the oil-stone ratio of SMA-13 asphalt mixture used by this article can be calculated should increase 0.1~0.3% or decrease 0.1~0.6% within appropriate porosity. Therefore, this article chooses the six different oil-stone ratios (5.5%, 5.7%, 5.9%, 6.1%, 6.3%, 6.5%) to performed the strength test to consider the influence of oil-stone ratio on asphalt mixture.

#### 2.2.2. Test Methods

To study the three-dimensional strength characteristics of asphalt mixtures, Huang [25] performed a triaxial failure test on a type of AC-13 asphalt mixture, and established a three-dimensional strength model characterized by tensile and compressive meridian, and failure strength envelope. The strength model which considering the cooperative destruction between each component is shown in Equation (1). However, the model is a complex nonlinear model due to contains five parameters and the form is complicated, which is not convenient for popularization and application.

(1) $$\begin{array}{c} \frac{\tau_{oct}^{c}}{f_{c}}=m\cdot \left(a-b\frac{\sigma_{oct}}{f_{c}}-c{\left(\frac{\sigma_{oct}}{f_{c}}\right)}^{2}\right)\\ \frac{\tau_{oct}^{t}}{f_{c}}=a-b\frac{\sigma_{oct}}{f_{c}}-c{\left(\frac{\sigma_{oct}}{f_{c}}\right)}^{2}\\ \tau_{oct}=\tau_{oct}^{t}-(\tau_{oct}^{t}-\tau_{oct}^{c})\sin^{n}(3\theta /2) \end{array}$$

where the $\sigma_{oct}$ is octahedral normal stress, $\tau_{oct}$ is octahedral shear stress, $f_{c}$ is uniaxial compression strength, θ is lode angle. The calculations are as follows:

(2) $$\sigma_{oct}=\frac{1}{3}(\sigma_{1}+\sigma_{2}+\sigma_{3})$$

(3) $$\tau_{oct}=\frac{1}{3}\sqrt{{\left(\sigma_{1}-\sigma_{2}\right)}^{2}+{\left(\sigma_{3}-\sigma_{2}\right)}^{2}+{\left(\sigma_{3}-\sigma_{1}\right)}^{2}}$$

(4) $$\theta =\arccos\left(\frac{2\sigma_{1}-\sigma_{2}-\sigma_{3}}{3\sqrt{2}\tau_{oct}}\right)$$

where $\sigma_{1}$, $\sigma_{2}$, $\sigma_{3}$ are each principal stress, where the unit is MPa, and the tension and compression are positive and negative respectively.

**Figure 5:**
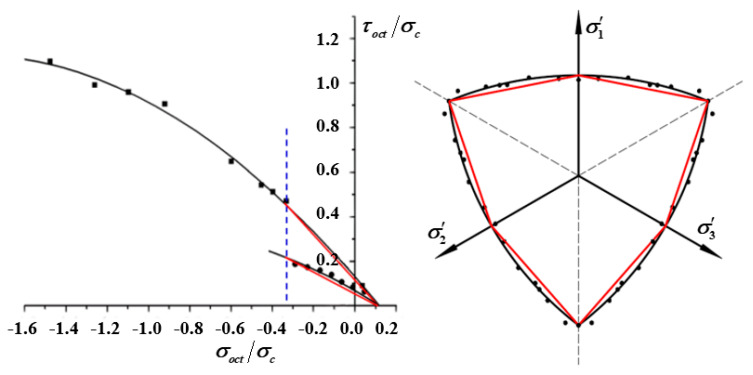
The nonlinear and linear strength models of asphalt mixture.

In pavement structure, the octahedron normal stress $\sigma_{oct}$ is usually less than 1/3 $f_{c}$. When the hydrostatic stress is less, the test results of the tensile and compressive meridian and the strength envelope can be characterized by straight lines. As shown in Figure 5, the failure criterion is established by linear regression. The design is safe, and the deviation is within 10%. By this way, the five-parameter non-linear three-dimensional strength model is simplified to a three-parameter linear strength model, and the model accuracy is guaranteed. The linear model can be established through unconfined compression, direct tensile and confining triaxial test which commonly used in road engineering. By substituting the above test results into the three sets of linear equations shown in Equation (5), the model parameters M, A, and B can be obtained.

(5) $$\frac{\tau_{oct}^{c}}{f_{c}}=M(A-B\frac{\sigma_{oct}}{f_{c}})\frac{\tau_{oct}^{t}}{f_{c}}=A-B\frac{\sigma_{oct}}{f_{c}}\tau_{oct}=\tau_{oct}^{t}-(\tau_{oct}^{t}-\tau_{oct}^{c})3\theta /\pi$$

#### 2.2.3. Test Schematic

**Figure 6:**
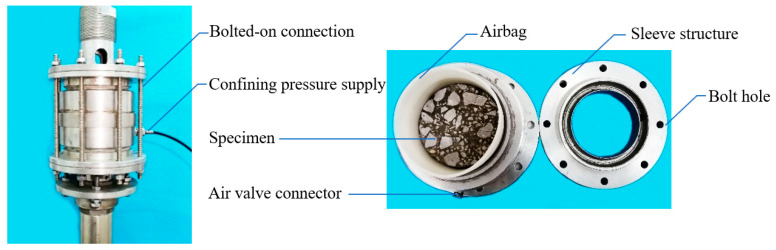
Strength test under various stress states.

It is known that the linear strength model can be obtained through uniaxial compressive, direct tensile and triaxial compressive tests. Before performing the direct/triaxial tensile test, clean the upper and lower contact surfaces of the pull plate and the test specimen and apply the adhesive, wait for the adhesive to fully cure, and then apply axial tensile stress to the test specimen until destroy the specimen. Through the self-developed airbag triaxial tester [25] to carry out the triaxial compression/tension test, as shown in Figure 6. Through the flexible airbag to apply the same compressive stress $\sigma_{1}$ and $\sigma_{2}$ ($\sigma_{2}$ and $\;\sigma_{3}$) in the horizontal direction, we then apply the axial compressive stress $\sigma_{13}$ ($\sigma_{1}$) to cause the failure of the specimen [27]. Before performing the uniaxial and triaxial compression tests, the upper and lower pressing plates and the specimen surface were wiped with Vaseline, and a cushion was added to reduce the friction between the specimen and the pressing plate. In addition, before the triaxial test, the inner wall of the airbag and the contact surface of the specimen should be rubbed with lubricating oil to lubricate the inner wall.

## 3. Results and Discussion

### 3.1. The Influence of Gradations and Asphalt Type in the Strength of Asphalt Mixture

**Figure 7:**
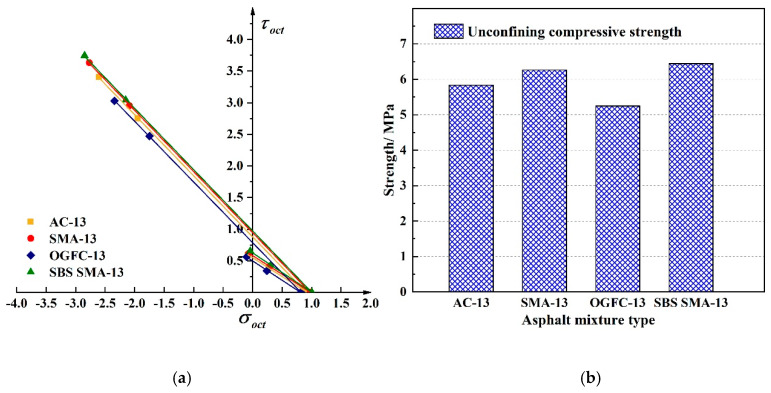
Linear compressive and tensile meridian under three type of gradation. (**a**) The compressive and tensile meridian under three type of gradation (**b**) The strength comparison between three type of gradation.

To analyze the effect of gradation on the strength of asphalt mixture, three type of asphalt mixtures were tested under optimal oil-stone ratio respectively, the test temperature was 20 °C and the loading rate was 2 mm/min. The average test results are shown in Table 4. Figure 7 shows the compressive and tensile strength comparison of three type of asphalt mixtures. Among the three gradations of asphalt mixture prepared by the 70# base bitumen, the compressive strength of SMA-13 asphalt mixture is higher than AC-13 asphalt mixture and OGFC-13 asphalt mixture, and the increase corresponding to AC-13 asphalt mixture and OGFC-13 asphalt mixture were 7.4% and 19.4%, respectively.

Based on the above strength test results, the simplified linear three-dimensional strength model can be established based on the following:

Tensile meridian:

(6) $$\frac{\tau_{oct}^{t}}{f_{c}}=A+B\frac{\sigma_{oct}}{f_{c}}\;R^{2}=0.95$$

Compressive meridian:

(7) $$\frac{\tau_{oct}^{c}}{f_{c}}=C\times \left(A+B\frac{\sigma_{oct}}{f_{c}}\right)\;R^{2}=0.94$$

Failure envelope curve:

(8) $$\tau_{oct}\left(\theta \right)=\tau_{oct}^{t}-\left(\tau_{oct}^{t}-\tau_{oct}^{c}\right)3\theta /\pi$$

where *A*, *B*, *C* is the fitting parameters, $R^{2}$ is the correlation coefficient. In addition, the model parameters are shown in Table 5.

As can be seen from Table 4 and Figure 7, AC-13, SMA-13, and OGFC-13 asphalt mixtures show differences in strength deformation. The SMA-13 asphalt mixture formulated by SBS modified bitumen has the best performance and the increasement to the SMA-13 asphalt mixture prepared by 70# base bitumen was 2.9%. The reason is mainly due to the fact that SMA-13 is the dense framework structure. Compared with AC-13, it has the higher asphalt content and less fine aggregate, and has a larger specific surface area, cohesion force and internal friction angle, which improves the strength and stability [28,29]. Compared with the suspend-dense structure AC-13, OGFC-13 is a typical skeleton-gap structure. It has a porosity of more than 18% and the fine aggregate is less than SMA-13. The space skeleton formed by the coarse aggregates close to each other cannot be effectively filled by the limited fine aggregates of the structure, the strength is less than AC-13. The use of modified SBS improves the adhesion between asphalt and aggregate, and improves the strength of the mixture [30,31]. Therefore, the SBS+SMA-13 asphalt mixture was used to study the strength under different oil-stone ratios, temperatures and loading rates.

### 3.2. Strength Test of Asphalt Mixture at Different Oil-Stone Ratios

To study the effect of oil-stone ratio to the strength of asphalt mixture, the SBS+SMA-13 asphalt mixture was performed, the loading rate was 2 mm/min, the test temperature was 20 °C, and the oil-stone ratios were 5.5%, 5.7%, 5.9%, 6.1%, 6.3%, 6.5%. The average test results are shown in Table 6.

Based on the above strength test results, the simplified linear three-dimensional strength model can be established based on the following:

Tensile meridian:

(9) $$\frac{\tau_{oct}^{t}}{a_{1}p^{2}+b_{1}p+c}=A+B\frac{\sigma_{oct}}{a_{1}p^{2}+b_{1}p+c}\;R^{2}=0.98$$

Compressive meridian:

(10) $$\frac{\tau_{oct}^{c}}{a_{1}p^{2}+b_{1}p+c}=C\times \left(A+B\frac{\sigma_{oct}}{a_{1}p^{2}+b_{1}p+c}\right)R^{2}=0.98$$

Failure envelope curve:

(11) $$\tau_{oct}\left(\theta \right)=\tau_{oct}^{t}-\left(\tau_{oct}^{t}-\tau_{oct}^{c}\right)3\theta /\pi$$

where A, B, C, $a_{1}$, $b_{1}$ and c are fitting parameters, and $f_{c}=a_{1}p^{2}+b_{1}p+c$, Where $\tau_{oct}^{c}$ and $\tau_{oct}^{t}$ are shear stress corresponding to the point on the compressive and tensile meridian respectively. Where *p* is oil-stone ratio, the unit of *p* is %. In addition, the model parameters are shown in Table 7.

**Figure 8:**
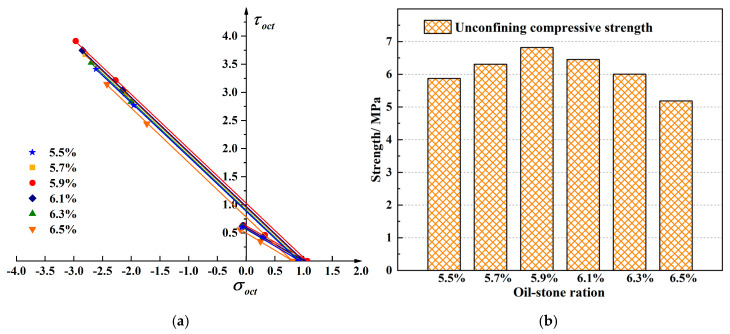
Linear compressive and tensile meridian under different oil-stone ratios. (**a**) The compressive and tensile meridian under six different oil-stone ratios (**b**) The strength comparison between six different oil-stone ratios.

**Figure 9:**
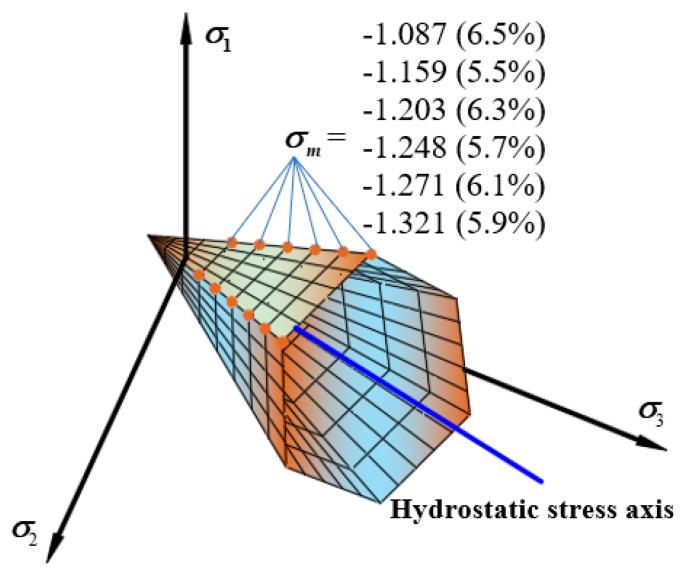
Linear failure envelope in π plane under different oil-stone ratios.

Figure 8 shows the tensile and compressive meridian under different oil-stone ratios. The ultimate strength of the asphalt mixture increased at first and then decreased with the increase of the oil-stone ratio. The strength change is obviously non-linear, and the strength value reaches the highest when it slightly lower than the optimal oil-stone ratio. The ultimate strength of the asphalt mixture increased at first and then decreased with the increase of the oil-stone ratio. The strength change is obviously non-linear, and the strength value reaches the highest when it slightly lower than the optimal oil-stone ratio. The reason is that the mixture has the bigger proportion of coarse aggregate and the smaller proportion fibers and fine aggregate, the specific surface area is large. As the asphalt content increases, the interspace in the mixture are continuously filled, the cohesion increases, and the strength increases. However, as the asphalt content continues to increase, more free asphalt is produced, filling the interspace in the mixture, it also pushes the minerals away from each other. Too much free asphalt causes the cohesion to decrease. The obtained maximum three-dimensional strength corresponds to the oil-stone ratio which is slightly lower than the optimal oil-stone ratio, with the oil-stone ratio value of 5.9% instead of the Marshall optimal oil-stone ratio of 6.1%. The failure envelope under different oil-stone ratios in π plane is shown in Figure 9.

### 3.3. Strength Test of Asphalt Mixture at Different Temperatures

To study the effect of temperatures to the strength of asphalt mixture, the SBS+SMA-13 asphalt mixture was performed, the test temperatures were 5 °C, 10 °C, 15 °C, 20 °C, 25 °C, 30 °C, the loading rate was 2 mm/min, the oil-stone ratio was 5.9%. The average test results are shown in Table 8.

Based on the above strength test results, the simplified linear three-dimensional strength model can be established based on the following:

Tensile meridian:

(12) $$\frac{\tau_{oct}^{t}}{a_{2}+b_{2}t}=A+B\frac{\sigma_{oct}}{a_{2}+b_{2}t}\;\;R^{2}=0.98$$

Compressive meridian:

(13) $$\frac{\tau_{oct}^{t}}{a_{2}+b_{2}t}=C\times \left(A+B\frac{\sigma_{oct}}{a_{2}+b_{2}t}\right)\;R^{2}=0.98$$

Failure envelope curve:

(14) $$\tau_{oct}\left(\theta \right)=\tau_{oct}^{t}-\left(\tau_{oct}^{t}-\tau_{oct}^{c}\right)3\theta /\pi$$

where *A*, *B*, *C*, $a_{2}$ and $b_{2}$ are fitting parameters, and $f_{c}=a_{2}+b_{2}t$, *t* is temperature. In addition, the model parameters are shown in Table 9.

**Figure 10:**
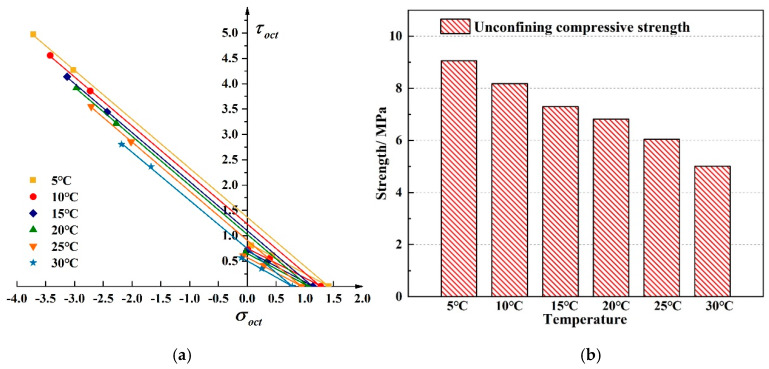
Linear compressive and tensile meridian under different temperature. (**a**) The compressive and tensile meridian under six different temperatures (**b**) The strength comparison between six different temperatures.

**Figure 11:**
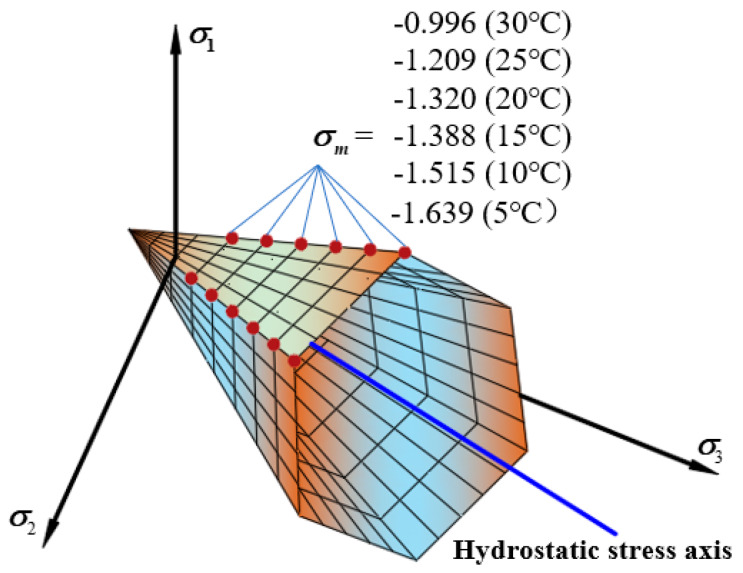
Linear failure envelope in π plane under different temperatures.

Figure 10 shows the linear compressive and tensile meridians under different temperatures. The octahedral tensile and compressive strength of the asphalt mixture decreases with increasing temperature. Uniaxial compressive strength exhibits a linear characteristic with temperature. From 5 °C to 30 °C, the uniaxial compressive strength of the mixture decreased by 40%. The change in temperature makes the asphalt molecules flow faster, and the contact angle between the asphalt and the aggregate becomes smaller [15], which in turn makes the asphalt mixture show a decrease in strength; the performance is reduced due to the high-temperature dependence of the mixture will continue. Selecting asphalt with better temperature stability can effectively maintain long-term service of asphalt pavement. The failure envelope under different temperatures in π plane is shown in Figure 11.

### 3.4. Strength Test of Asphalt Mixture at Different Loading Rates

To study the effect of loading rates to the strength of asphalt mixture, the SBS+SMA-13 asphalt mixture was performed, the test temperatures were 1 mm/min, 2 mm/min, 3 mm/min, 4 mm/min, 5 mm/min, the test temperature was 20 °C, the oil-stone ratio was 5.9%. The average test results are shown in Table 10.

Based on the above strength test results, the simplified linear three-dimensional strength model can be established based on the following:

Tensile meridian:

(15) $$\frac{\tau_{oct}^{t}}{a_{3}+b_{3}v}=A+B\frac{\sigma_{oct}}{a_{3}+b_{3}v}\;R^{2}=0.98$$

Compressive meridian:

(16) $$\frac{\tau_{oct}^{c}}{a_{3}+b_{3}v}=C\times \left(A+B\frac{\sigma_{oct}}{a_{3}+b_{3}v}\right)\;R^{2}=0.98$$

Failure envelope curve:

(17) $$\tau_{oct}\left(\theta \right)=\tau_{oct}^{t}-\left(\tau_{oct}^{t}-\tau_{oct}^{c}\right)3\theta /\pi$$

where *A*, *B*, *C*, $a_{3}$ and $b_{3}$ are fitting parameters, and $f_{c}=a_{3}+b_{3}v$. 

where v is loading rates, the unit of v is mm/min. In addition, the model parameters are shown in Table 11.

**Figure 12:**
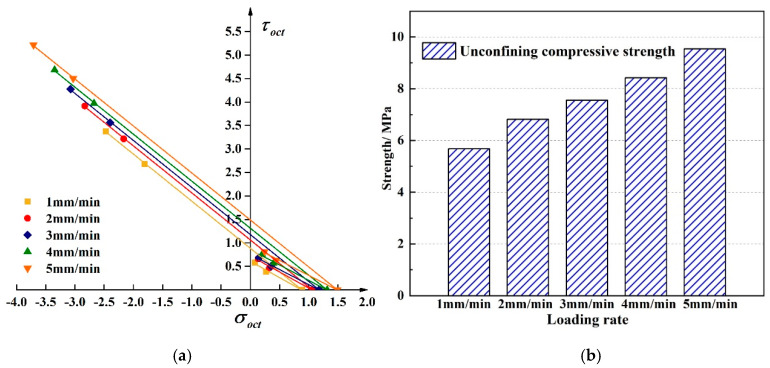
Linear compressive and tensile meridian under different loading rates. (**a**) The compressive and tensile meridian under six different temperatures (**b**) The strength comparison between six different temperatures.

**Figure 13:**
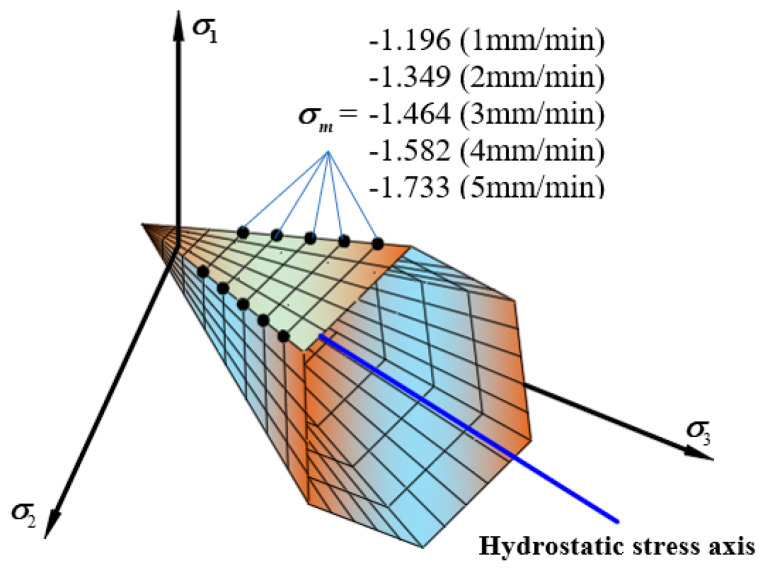
Linear failure envelope in π plane under different loading rates.

Figure 12 shows the tensile and compressive meridian under different loading rates. The results show in the test loading rates range, as the loading rate increased, the tensile and compressive strengths showed an increasing trend. From 1 mm/min to 5 mm/min, the uniaxial compressive strength increased by 68%. The change of the loading rate is the change of the loading time. When the loading rate increases, the time from when the asphalt mixture starts to crack to the failure of the specimen gradually decreases. The asphalt mixture cannot show a complete mechanical response [32], so it shows a reduction in deformation and the strength increases. The failure envelope under different loading rates in π plane is shown in Figure 13.

The unified calculation model for the three-dimensional strength of asphalt mixtures considering the effects of temperature, loading speed, and oil-stone ratio:

Tensile meridian:

(18) $$\frac{\tau_{oct}^{t}}{f_{c,\left(t,v,p\right)}}=A+B\frac{\sigma_{oct}}{f_{c,\left(t,v,p\right)}}\;R^{2}=0.98$$

Compressive meridian:

(19) $$\frac{\tau_{oct}^{c}}{f_{c,\left(t,v,p\right)}}=C\times \left(A+B\frac{\sigma_{oct}}{f_{c,\left(t,v,p\right)}}\right)R^{2}=0.98$$

Failure envelope curve:

(20) $$\tau_{oct}\left(\theta \right)=\tau_{oct}^{t}-\left(\tau_{oct}^{t}-\tau_{oct}^{c}\right)3\theta /\pi$$

where $f_{c,\left(t,v,p\right)}$ is the uniaxial compressive strength function of SMA-13 asphalt mixture, and the model parameters are shown in the above.

**Figure 14:**
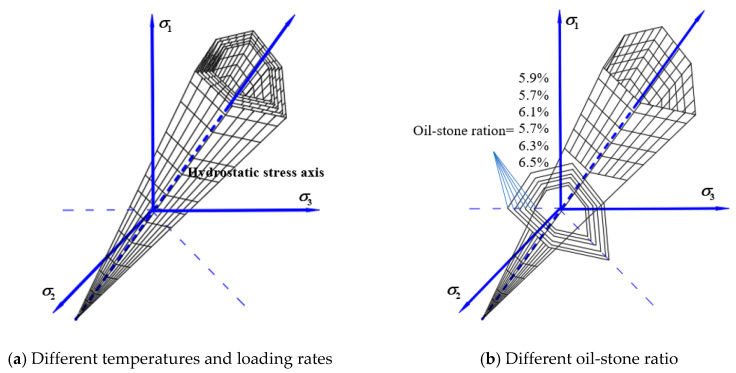
Linear failure criterion under different test conditions.

The change law of linearized three-dimensional failure envelope surface can be obtained with the temperatures, loading rates, and the oil-stone ratios. Figure 14 shows the linear failure criterion in the $\sigma_{oct}-\tau_{oct}$ space. The middle symmetry axis in the three-dimensional failure envelope surface is the hydrostatic stress axis as shown in Figure 14, the compressive meridian is composed of all points of the lodes angle 60°, and the tensile meridian is composed of all points of the lode angle 0°. From the above, in the temperatures range of 5 °C~30 °C and the loading rates range of 1 mm/min~5 mm/min, the three-dimensional basic strength of the asphalt mixture changes linearly with the test conditions. As the temperature increases, the enveloping surface of the asphalt mixture failure shrinks continuously, and as the loading rate increases, the enveloping surface continues to expand until the strength limit is reached. The change of the three-dimensional failure envelope surface with the oil-stone ratio is a kind of non-linearity parabolic form, and the envelope surface appears to expand first and then contract.

### 3.5. The Influence of Different Factors to the Strength of Asphalt Mixture

To quantitatively reveal the effects of four factors: temperature, loading speed, asphalt type, and oil-stone ratio on the three-dimensional strength characteristics of SMA-13 asphalt mixture, a three-factor three-level orthogonal experiment study was carried out [33]. This paper used two sets of orthogonal tests $L_{9}\left(3^{4}\right)$ of SBS modified asphalt and base asphalt respectively. The orthogonal test schemes are shown in Table 12, where N is the test number, T is the temperature, V is the loading speed, O is the oil-stone ratio, and UC is the uniaxial compression, TC is triaxial compression, UT is uniaxial tension.

In the orthogonal test, the range $R_{j}$ is an important index, and the value of this index increased with the greatest influence factors. Therefore, the influence of various factors on the strength of the asphalt mixture can be determined based on the range.

**Figure 15:**
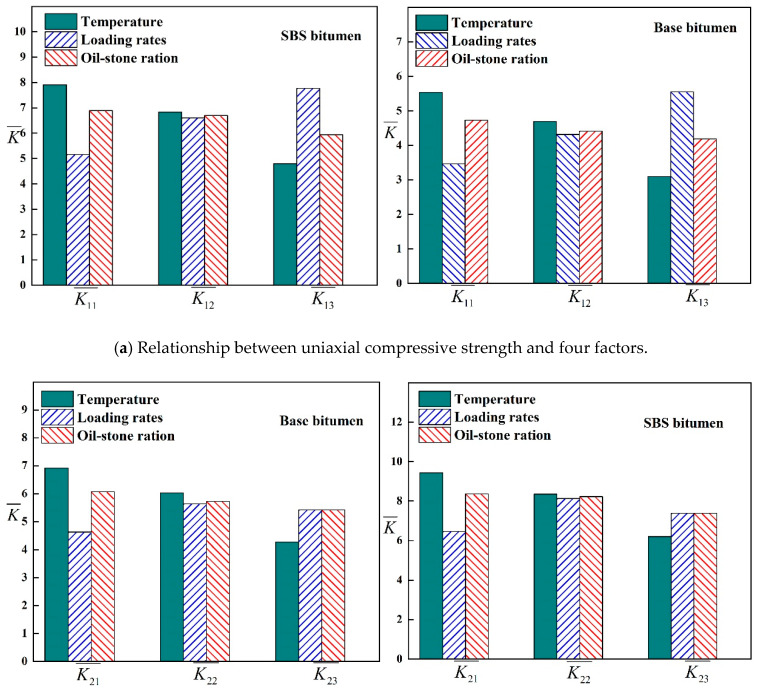

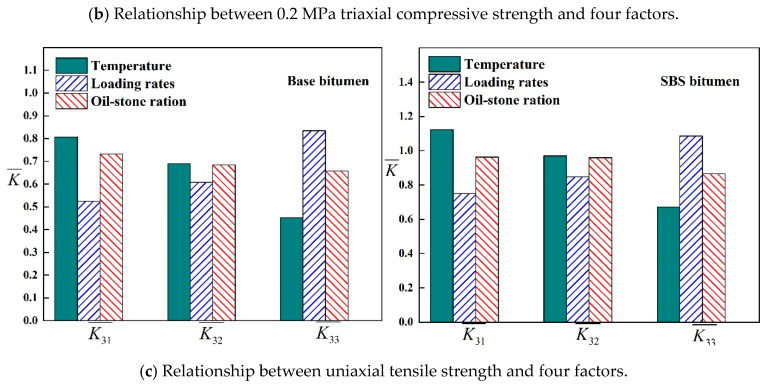
Three-factor range analysis.

$R_{j}=\max\left(\bar{K_{j1}},\bar{K_{j2}},\ldots ,\bar{K_{jm}}\right)-\min\left(\bar{K_{j1}},\bar{K_{j2}},\ldots ,\bar{K_{jm}}\right)$, where $K_{jm}$ is the sum of each test index under *j* column element and *m* level, $\bar{K_{jm}}$ is the average value of $K_{jm}$. From the value of $\bar{K_{jm}}$, the optimal combination can be judged by the superior level of the factor j and the combination of the superior levels of each factor. The orthogonal analysis results are shown in Table 13. The range analysis is shown in Figure 15.

It can be seen from Table 13 and Figure 15 that the greatest influence on the strength of the mixture is the asphalt type, the second most influence is the temperature, the less influence is the loading rate, and the least influence is the oil-stone ratio. This is because compared with matrix asphalt, SBS modified asphalt has a higher flexibility index [19], penetration and ductility are higher than Base asphalt, which improves the adhesion between asphalt and aggregate. As the temperature increases or the loading rate decreases, the strength of the mixture gradually decreases, which is mainly caused by the accelerated flow of the asphalt molecules and the decrease in the contact angle between the asphalt and the aggregate.

The combination of direct tensile, unconfined compression, and confining triaxial strength better characterizes the three-dimensional strength characteristics of the asphalt mixture, and the three-dimensional strength directly determines the mechanical properties of the mixture and the durability of the asphalt pavement structure. To give the mixture sufficient strength, it needs some reasonable control measures. Combining with the previous gradation and strength tests at different asphalt, oil-stone ratio, temperature, and loading rate, it is proposed here that SMA asphalt mixtures formulated with modified asphalt are preferentially used in pavement design. Under the requirements of the void ratio, the result is slightly lower than Marshall oil-stone ratio.

## 4. Conclusions

Uniaxial compression, direct tension, and confining triaxial tests were performed on SMA-13 asphalt mixture under different test conditions. A linearized three-dimensional strength model considering the influence of multiple factors was established. The effects of temperature, loading speed, and asphalt type on the three-dimensional strength were analyzed. Some conclusions are as follows:

(1) The single use of either the direct tensile, unconfined compression, or confining triaxial test reflects with difficulty the three-dimensional strength characteristics of the pavement material under three-dimensional stress. The combining of these strengths can give the three-dimensional strength of the asphalt mixture better characterized strength characteristics.
(2) Based on the linearized three-dimensional strength model, a unified three-dimensional strength calculation method that takes into account the effects of temperature, loading speed, and oil-stone ratio is proposed. It is revealing that the strength of the mixture decreases with increasing temperature, increases with the increase of loading rate, and increases first then decreases with increasing oil-stone ratio. It provides a reference for the estimation of the three-dimensional strength of the mixture.
(3) Two sets of three-factor three-level orthogonal tests were carried out on the SMA-13 asphalt mixture. The effects of asphalt type, temperature, loading rate, and oil-stone ratios were analyzed. The results show that the above test conditions have an impact on the strength of the mixture in order. Two main measures are proposed to improve the strength of the asphalt mixture: (1) use the modified asphalt and high-temperature stability high-quality asphalt; (2) use the lower oil-stone ratio than the Marshall optimal oil-stone ratio.

Although this article has established a multi-factor impact criterion for the three-dimensional strength of asphalt mixtures, with the intensification of heavy-duty traffic and overloading, hot and freezing weather in northern and southern China has increased, and severe weather phenomena such as acid rain have occurred frequently. It is necessary to analyze the strength of the complex stress state of the mixture under multiple harsh environments, and carry out research on new materials such as nano-modified asphalt mixtures to comprehensively improve the mechanical properties of the mixture. It is recommended to use SMA and a high-performance modified asphalt mixture for subsequent research in the future.

## Figures and Tables

**Table 1:** The density of basalt aggregate at all levels.

Sieve Size (mm)	Apparent Density (g/cm^3^)	Standard Requirement	Test Methods
13.2	2.680	≥2.6	T0304-2004
9.5	2.682	≥2.6	T0304-2004
4.75	2.673	≥2.6	T0304-2004
2.36	2.660	≥2.5	T0328-2004
1.18	2.651	≥2.5	T0328-2004
0.6	2.617	≥2.5	T0328-2004
0.3	2.604	≥2.5	T0328-2004
0.15	2.594	≥2.5	T0328-2004
0.075	2.627	≥2.5	T0328-2004
Ore powder	2.763	≥2.5	T0352-2004

**Table 2:** The key index of two type of asphalt.

Test	Test Methods	Test Results		Standard Requirement	
		SBS Asphalt	Base Asphalt	SBS Asphalt	Base Asphalt
Penetration (25 °C, 100 g, 5 s)	T0604-2004	56.1	69	40~60	60–80
Penetration Index	T0604-2004	0.533	−0.78	≥0	−1.5~+1.0
Softening point (°C)	T0606-2004	80	48	≥60	≥46
Ductility, cm	T0605-2004	32(5 °C)	22.8(10 °C)	≥20	≥20
Kinematic viscosity 135 °C (Pa·s)	T0625-2004	2.30	-	≤3	-
Dynamic viscosity 60 °C (Pa·s)	T0620-2004	-	192	-	≥180
Solubility (%)	T0607-2004	99.9	99.8	≥99	≥99.5

**Table 3:** The Marshall test results.

Mixture Type		VV (%)	Oil-Stone Ratio (%)	VMA (%)	VFA (%)	MS (KN)	FL (mm)
AC-13	SBS	4.5	5.7	15.2	70.4	15.7	2.7
	Base	4.2	5.2	14.6	71.2	14.1	2.4
SMA-13	SBS	4.7	6.1	19.8	76.3	6.3	–
	Base	4.3	5.7	17.2	75	5.8	3.7
OGFC-13	SBS	19.5	5.4	12.8	–	6.9	–
	Base	20.9	4.9	10.5	–	4.1	–

**Table 4:** Test results of three type of asphalt mixture under Marshall optimum oil-stone ratio.

Asphalt Type	Mixture Type	$\sigma_{1}/\mathrm{MPa}$	$\sigma_{2}/\mathrm{MPa}$	$\sigma_{3}/\mathrm{MPa}$	$\sigma_{oct}/f_{c}$	$\tau_{oct}/f_{c}$	θ/°
Base asphalt	AC-13	0	0	−5.835	−0.333	0.471	60
		−0.2	−0.2	−7.417	−0.447	0.583	60
		0.845	0	0	0.0483	0.068	0
		0.748	−0.5	−0.5	−0.014	0.101	0
	SMA-13	0	0	−6.265	−0.333	0.471	60
		−0.2	−0.2	−7.905	−0.442	0.58	60
		0.885	0	0	0.0471	0.067	0
		0.806	−0.5	−0.5	−0.01	0.098	0
	OGFC-13	0	0	−5.245	−0.333	0.471	60
		−0.2	−0.2	−6.625	−0.446	0.577	60
		0.727	0	0	0.0462	0.065	0
		0.698	−0.5	−0.5	−0.019	0.108	0
SBS asphalt	SMA-13	0	0	−6.449	−0.333	0.471	60
		−0.2	−0.2	−8.146	−0.442	0.581	60
		0.918	0	0	0.0474	0.067	0
		0.883	−0.5	−0.5	−0.006	0.101	0

**Table 5:** Fitting parameters of linear model under different gradation.

Modeling Parameters	*A*	*B*	*C*
Fitting Results	0.1	−0.64	1.512

**Table 6:** Test results of asphalt mixture under different oil-stone ratio.

Oil-Stone Ratio	$\sigma_{1}/\mathrm{MPa}$	$\sigma_{2}/\mathrm{MPa}$	$\sigma_{3}/\mathrm{MPa}$	$\sigma_{oct}/f_{c}$	$\tau_{oct}/f_{c}$	θ/°
5.5%	0	0	−5.873	−0.33	0.47	60
	−0.2	−0.2	−7.441	−0.45	0.58	60
	0.885	0	0	0.05	0.07	0
	0.777	−0.5	−0.5	−0.01	0.1	0
5.7%	0	0	−6.306	−0.333	0.471	60
	−0.2	−0.2	−7.991	−0.443	0.582	60
	0.905	0	0	0.0478	0.068	0
	0.795	−0.5	−0.5	−0.011	0.097	0
5.9%	0	0	−6.819	−0.333	0.471	60
	−0.2	−0.2	−8.501	−0.435	0.574	60
	0.981	0	0	0.0479	0.068	0
	0.853	−0.5	−0.5	−0.007	0.094	0
6.1%	0	0	−6.449	−0.333	0.471	60
	−0.2	−0.2	−8.146	−0.442	0.581	60
	0.918	0	0	0.0474	0.067	0
	0.826	−0.5	−0.5	−0.009	0.097	0
6.3%	0	0	−6.005	−0.333	0.471	60
	−0.2	−0.2	−7.685	−0.449	0.588	60
	0.864	0	0	0.048	0.068	0
	0.774	−0.5	−0.5	−0.013	0.1	0
6.5%	0	0	−5.183	−0.333	0.471	60
	−0.2	−0.2	−6.868	−0.467	0.606	60
	0.743	0	0	0.0478	0.068	0
	0.689	−0.5	−0.5	−0.02	0.108	0

**Table 7:** Fitting parameters of linear model under different oil-stone ratio.

Asphalt Type	*A*	*B*	*C*	$a_{1}$	$b_{1}$	c
SBS Modified Asphalt	0.1	−0.64	1.512	−4.51	53.45	−151.69

**Table 8:** Test results of asphalt mixture under different temperatures.

Temperature	$\sigma_{1}/\mathrm{MPa}$	$\sigma_{2}/\mathrm{MPa}$	$\sigma_{3}/\mathrm{MPa}$	$\sigma_{oct}/f_{c}$	$\tau_{oct}/f_{c}$	θ/°
5 °C	0	0	−9.059	−0.333	0.471	60
	−0.2	−0.2	−10.74	−0.41	0.548	60
	1.304	0	0	0.048	0.068	0
	1.213	−0.5	−0.5	0.0078	0.089	0
10 °C	0	0	−8.177	−0.333	0.471	60
	−0.2	−0.2	−9.862	−0.418	0.557	60
	1.174	0	0	0.0479	0.068	0
	1.038	−0.5	−0.5	0.0015	0.089	0
15 °C	0	0	−7.301	−0.333	0.471	60
	−0.2	−0.2	−8.979	−0.428	0.567	60
	1.052	0	0	0.048	0.068	0
	0.987	−0.5	−0.5	−0.0006	0.096	0
20 °C	0	0	−6.819	−0.333	0.471	60
	−0.2	−0.2	−8.501	−0.435	0.574	60
	0.981	0	0	0.0479	0.068	0
	0.905	−0.5	−0.5	−0.005	0.097	0
25 °C	0	0	−6.049	−0.333	0.471	60
	−0.2	−0.2	−7.727	−0.448	0.587	60
	0.872	0	0	0.048	0.068	0
	0.785	−0.5	−0.5	−0.012	0.1	0
30 °C	0	0	−5.012	−0.333	0.471	60
	−0.2	−0.2	−6.145	−0.435	0.559	60
	0.747	0	0	0.0497	0.07	0
	0.698	−0.5	−0.5	−0.02	0.113	0

**Table 9:** Fitting parameters of linear model under different temperatures.

Asphalt Type	*A*	*B*	*C*	$a_{2}$	$b_{2}$
SBS Modified Asphalt	0.1	−0.64	1.512	9.78	−0.155

**Table 10:** Test results of asphalt mixture under different loading rates.

Loading Rates	$\sigma_{1}/\mathrm{MPa}$	$\sigma_{2}/\mathrm{MPa}$	$\sigma_{3}/\mathrm{MPa}$	$\sigma_{oct}/f_{c}$	$\tau_{oct}/f_{c}$	θ/°
1 mm/min	0	0	−5.678	0.317	−0.471	60
	−0.2	−0.2	−7.359	0.435	−0.594	60
	0.817	0	0	−0.05	−0.068	0
	0.732	−0.5	−0.5	−0.01	−0.102	0
2 mm/min	0	0	−6.819	0.317	−0.471	60
	−0.2	−0.2	−8.501	0.415	−0.574	60
	0.981	0	0	−0.05	−0.068	0
	0.905	−0.5	−0.5	−0.02	−0.097	0
3 mm/min	0	0	−7.555	0.317	−0.471	60
	−0.2	−0.2	−9.268	0.407	−0.566	60
	1.064	0	0	−0.05	−0.066	0
	0.943	−0.5	−0.5	−0.02	−0.09	0
4 mm/min	0	0	−8.422	0.317	−0.471	60
	−0.2	−0.2	−10.13	0.398	−0.556	60
	1.192	0	0	−0.05	−0.067	0
	1.107	−0.5	−0.5	−0.02	−0.09	0
5 mm/min	0	0	−9.546	0.317	−0.471	60
	−0.2	−0.2	−11.26	0.389	−0.546	60
	1.339	0	0	−0.05	−0.066	0
	1.231	−0.5	−0.5	−0.03	−0.085	0

**Table 11:** Fitting parameters of linear model under different loading rates.

Asphalt type	*A*	*B*	*C*	*a_3_*	*b_3_*
SBS modified asphalt	0.1	−0.64	1.56	4.803	0.934

**Table 12:** Design plane of orthogonal test.

Asphalt Type	N	T (°C)	V mm/min	O(%)	UC	TC	UT
Base bitumen	1	5	1	5.7	−4.598	−5.925	0.728
	2	5	3	5.9	−5.449	−6.844	0.849
	3	5	5	6.1	−6.556	−7.983	0.999
	4	15	1	5.9	−3.848	−5.08	0.624
	5	15	3	6.1	−4.071	−5.39	0.651
	6	15	5	5.7	−6.165	−7.606	0.929
	7	25	1	6.1	−1.938	−2.88	0.325
	8	25	3	5.7	−3.432	−4.673	0.543
	9	25	5	5.9	−3.929	−5.239	0.577
SBS bitumen	1	5	1	5.7	−6.595	−7.947	0.953
	2	5	3	5.9	−8.352	−9.939	1.195
	3	5	5	6.1	−8.778	−10.4	1.249
	4	15	1	5.9	−5.848	−7.23	0.858
	5	15	3	6.1	−6.014	−7.563	0.89
	6	15	5	5.7	−8.62	−10.27	1.185
	7	25	1	6.1	−3.028	−4.196	0.456
	8	25	3	5.7	−5.458	−6.88	0.752
	9	25	5	5.9	−5.903	−7.511	0.824

**Table 13:** The results of orthogonal test.

	Base Asphalt			SBS Modified Asphalt		
	T	V	O	T	V	O
$\bar{K_{11}}$	5.534	3.462	4.732	7.908	5.157	6.891
$\bar{K_{12}}$	4.695	4.317	4.409	6.827	6.608	6.701
$\bar{K_{13}}$	3.1	5.55	4.188	4.796	7.767	5.94
$\bar{K_{21}}$	6.917	4.628	6.068	9.427	6.457	8.365
$\bar{K_{22}}$	6.025	5.636	5.721	8.353	8.127	8.227
$\bar{K_{23}}$	4.264	5.418	5.418	6.196	7.385	7.385
$\bar{K_{31}}$	0.806	0.525	0.733	1.124	0.75	0.963
$\bar{K_{32}}$	0.69	0.608	0.684	0.97	0.847	0.959
$\bar{K_{33}}$	0.452	0.835	0.658	0.672	1.086	0.865
$R_{1}$	2.434	2.088	0.543	3.112	2.61	0.951
$R_{2}$	2.654	1.008	0.65	3.231	1.67	0.98
$R_{3}$	0.354	0.311	0.075	0.451	0.336	0.098

## References

[1] Cai J., Pei J., Luo Q., Zhang J., Li R., Chen X. (2017). Comprehensive service properties evaluation of composite grouting materials with high-performance cement paste for semi-flexible pavement. Constr. Build. Mater..

[2] Contreras M., Teixeira S.R., Lucas M.C., Lima L.C.N., Cardoso D.S.L., da Silva G.A.C., Gregorio G.C., de Souza A.E., dos Santos A. (2016). Recycling of construction and demolition waste for producing new construction material (Brazil case-study). Constr. Build. Mater..

[3] Guan H.-X., Wang H.-Q., Liu H., Yan J.-J., Lin M. (2018). The Effect of Intermediate Principal Stress on Compressive Strength of Different Cement Content of Cement-Stabilized Macadam and Different Gradation of AC-13 Mixture. Appl. Sci..

[4] Li N., Molenaar A.A.A., Pronk A.C., van de Ven M.F.C., Wu S. (2015). Application of the partial healing model on laboratory fatigue results of asphalt mixture. Constr. Build. Mater..

[5] Cheng J., Qian X. (2015). Temperature-dependent viscoelastic model for asphalt concrete using discrete rheological representation. Constr. Build. Mater..

[6] Li J., Zhang J., Qian G., Zheng J., Zhang Y. (2019). Three-dimensional simulation of aggregate and asphalt mixture using parameterized shape and size gradation. J. Mater. Civ. Eng..

[7] Moreno-Navarro F., Sol-Sanchez M., Rubio-Gamez M.C., Segarra-Martinez M. (2014). The use of additives for the improvement of the mechanical behavior of high modulus asphalt mixes. Constr. Build. Mater..

[8] Kim J., Koh C. (2012). Development of a Predictive System for Estimating Fatigue Life of Asphalt Mixtures Using the Indirect Tensile Test. J. Transp. Eng. ASCE.

[9] Kim M., Mohammad L.N., Phaltane P., Elseifi M.A. (2017). Density and SCB measured fracture resistance of temperature segregated asphalt mixtures. Int. J. Pavement Res. Technol..

[10] Safavizadeh S.A., Kim Y.R. (2017). DIC Technique to Investigate Crack Propagation in Grid-Reinforced Asphalt Specimens. J. Mater. Civ. Eng..

[11] Falchetto A.C., Moon K.H., Wang D., Riccardi C., Wistuba M.P. (2018). Comparison of low-temperature fracture and strength properties of asphalt mixture obtained from IDT and SCB under different testing configurations. Road Mater. Pavement Des..

[12] Kim D., Kim Y.R. (2017). Development of Stress Sweep Rutting (SSR) test for permanent deformation characterization of asphalt mixture. Constr. Build. Mater..

[13] Lee H.S. (2015). Application of the Viscoelastic Continuum Damage Mechanics to Asphalt Mixtures under Indirect Tensile Load. J. Eng. Mech..

[14] Pszczola M., Jaczewski M., Szydlowski C. (2019). Assessment of Thermal Stresses in Asphalt Mixtures at Low Temperatures Using the Tensile Creep Test and the Bending Beam Creep Test. Appl. Sci..

[15] Yao H., Dai Q., You Z., Zhang J., Lv S., Xiao X. (2019). Evaluation of contact angle between asphalt binders and aggregates using Molecular Dynamics (MD) method. Constr. Build. Mater..

[16] Si W., Li N., Ma B., Tian Y.-X., Zhou X.-Y. (2016). Temperature response to tensile characteristics of the hot asphalt mixtures. Ksce J. Civ. Eng..

[17] Habeeb H., Chandra S., Nashaat Y. (2014). Estimation of moisture damage and permanent deformation in asphalt mixture from aggregate gradation. Ksce J. Civ. Eng..

[18] Liu H., Yang X., Jiang L., Lv S., Huang T., Yang Y. (2020). Fatigue-creep damage interaction model of asphalt mixture under the semi-sine cycle loading. Construction and Building Materials.

[19] Ranieri M., Celauro C. (2018). Improvement of high modulus asphalt mixtures with average quality aggregate and bitumen by application of polymeric additives. Constr. Build. Mater..

[20] Ge D., You Z., Chen S., Liu C., Gao J., Lv S. (2019). The performance of asphalt binder with trichloroethylene: Improving the efficiency of using reclaimed asphalt pavement. J. Clean. Prod..

[21] Sreedhar S., Coleri E. (2018). Effects of Binder Content, Density, Gradation, and Polymer Modification on Cracking and Rutting Resistance of Asphalt Mixtures Used in Oregon. J. Mater. Civ. Eng..

[22] Fang M., Park D., Singuranayo J.L., Chen H., Li Y. (2019). Aggregate gradation theory, design and its impact on asphalt pavement performance: A review. Int. J. Pavement Eng..

[23] Huang T., Pan Q.X., Jin J., Zheng J.L., Wen P.H. (2019). Continuous constitutive model for bimodulus materials with meshless approach. Applied Mathematical Modelling.

[24] Zhou X.-P., Huang X.-C., Berto F. (2018). A three-dimensional long-term strength criterion of rocks based on micromechanical method. Theor. Appl. Fract. Mech..

[25] Huang T., Zheng J.L., Lv S.T., Zhang J.H., Wen P.H., Bailey C.G. (2018). Failure criterion of an asphalt mixture under three-dimensional stress state. Constr. Build. Mater..

[26] Airey G.D., Hunter A.E., Collop A.C. (2008). The effect of asphalt mixture gradation and compaction energy on aggregate degradation. Constr. Build. Mater..

[27] Huang T., Qi S., Yang M., Lv S., Liu H., Zheng J. (2018). Strength Criterion of Asphalt Mixtures in Three-Dimensional Stress States under Freeze-Thaw Conditions. Appl. Sci..

[28] Luo S., Qian Z., Yang X., Wang H. (2017). Design of gussasphalt mixtures based on performance of gussasphalt binders, mastics and mixtures. Constr. Build. Mater..

[29] Zhao Y., Jiang L., Zhou L., Jiang J. (2017). Heterogeneous fracture simulation of asphalt mixture under beam bending test with cohesive zone modeling. Transportation Research Congress.

[30] Hasan M.R.M., You Z.P., Satar M., Warid M.N.M., Kamaruddin N.H.M., Ge D.D., Zhang R. (2018). Effects of titanate coupling agent on engineering properties of asphalt binders and mixtures incorporating lldpe-caco3 pellet. Appl. Sci..

[31] Pirmohammad S., Ayatollahi M.R. (2014). Fracture resistance of asphalt concrete under different loading modes and temperature conditions. Constr. Build. Mater..

[32] Lv S., Xia C., Liu C., Zheng J., Zhang F. (2019). Fatigue equation for asphalt mixture under low temperature and low loading frequency conditions. Constr. Build. Mater..

[33] Cheng Y., Li L., Zhou P., Zhang Y., Liu H. (2019). Multi-Objective Optimization Design and Test of Compound Diatomite and Basalt Fiber Asphalt Mixture. Materials.

